# Treatment of advanced stage osteochondrosis dissecans in the adolescent elbow using a hyaloronic acid-based scaffold: a case series of 5 patients

**DOI:** 10.1007/s00402-021-03773-8

**Published:** 2021-02-04

**Authors:** Sebastian Farr, Matthias Pallamar, Theresa Eder, Rudolf Ganger

**Affiliations:** 1 Orthopedic Hospital Speising, Department of Pediatric Orthopaedics and Adult Foot and Ankle Surgery, Medical University Vienna, Speisingerstrasse 109, 1130 Vienna, Austria; 2 Medical University Vienna, Vienna, Austria

**Keywords:** Osteochondrosis dissecans, Osteochondritis, AMIC, Bone graft, Pediatric elbow

## Abstract

**Introduction:**

Osteochondrosis dissecans (OCD) is considered to be one of the main causes for pain, discomfort and morbidity in the pediatric elbow joint. Few treatment options, such as microfracture or autologous transplantation, of osteochondral bone grafts have been described to address advanced OCD. The aim of this retrospective case series is to present preliminary clinical and radiologic findings following advanced stage OCD repair using a novel combination of a hyaluronic acid-based scaffold with autologous iliac crest bone grafting.

**Materials and methods:**

Five adolescents, who underwent treatment of OCD (grade 3 or 4 according to Nelson) using a combination technique of defect debridement, transplantation of cancellous iliac crest bone and application of a HYALOFAST® membrane (Anika Therapeutics S.r.L., Italy), were re-assessed using clinical and radiologic examinations (defect diameter, depth, sclerosis, congruency, fragmentation, dissection, radiolucency, growth plate status; MRI) after a minimum of 2 years (mean, 34 months; range, 24–45) postoperatively. Dedicated outcome scores (Numeric Rating Scale [NRS], Pediatric Outcome Data Collection Instrument [PODCI], Mayo Elbow Performance Score [MEPS], and Timmerman-Andrews Score [TIMM] were collected.

**Results:**

All patients reported a NRS score of 0. The mean total TIMM, MEPS and PODCI (Global Functioning Scale) scores were 189 (range 165–200), 94 (range, 70–100), and 92 (range 83–98; normative score 47; range 35–55), respectively, indicating good to excellent clinical outcomes. The radiographic analysis showed overall improvements with regard to OCD width and depth reduction (35%, − 27–100%; 52%, 4–100%), but full resolution in only 2 of 5 cases. Elbow motion improved slightly after surgery. No complications were noted.

**Conclusion:**

This study showed promising clinical short- to mid-term results in adolescent patients with advanced OCD using a novel surgical treatment combination. Radiographic results showed partial healing; hence, residual changes should be monitored over a longer period.

## Introduction

Osteochondrosis dissecans (OCD) incidence has been reported to be around 2.2 per 100,000 (3.4 per 100,000 for 12- to 19-year-olds) and may be more prominent in athletes, who are particularly prone to repetitive loading in the radiocapitellar joint [1, 2]. Male gender and extreme obesity were found to be further risk factors for the development of elbow OCD [1, 3]. To date, current literature indicates that repetitive stress and altered radiocapitellar mechanics are likely the main causes for OCD besides unknown environmental, endocrinologic and genetic factors [4, 5]. Untreated OCD may progress to degenerative, osteoarthritic-like changes of the cartilage [6]. Therefore, thorough evaluation of the painful elbow joint in young children and athletes is mandatory to avoid delayed diagnosis and progression to surgery [7, 8].

Several different treatment strategies have been applied in both immature and adult patient cohorts. In general, treatment is guided by the stage of OCD and whether the OCD lesion is stable or unstable. In general, early-stage pediatric OCD (with open growth plates) can be successfully handled by conservative means, such as rest and sports restriction [9]. However, previous reports have shown that 55% of elbow OCD cases still progress to surgical treatment [8]. The options for therapy-resistant and/or advanced OCD include fragment resection alone [10], arthroscopy with antegrade or retrograde drilling [11–13], fixation of the fragment using bioabsorbable implants [14, 15] or osteochondral autografts (OAG [16, 17]), and corrective osteotomy of the distal humerus [18, 19]. In cases with OCD grade 4 (loose body), it is unavoidable to create a new subchondral bone stock and cartilage layer in the defect area [20]. This can be achieved by either autologous transplantation of OAG, which has the potential disadvantage of donor site morbidity (e.g. in the knee joint), or by application of tissue scaffolds [21, 22]. The latter have been shown to be effective in achieving a stable, regenerative cartilage-like tissue [22]. Several reports in the adult literature have highlighted the great healing potential of the so-called autologous matrix-induced chondrogenesis (AMIC) technique in the knee and ankle joint [23–25]. Of note, no such reports have so far been published with regard to an immature patient cohort.

The aim of this retrospective case series is to present our preliminary clinical and radiologic findings following advanced stage OCD repair using a novel combination of a hyaluronic acid-based scaffold with autologous cancellous bone grafting. We sought to determine whether this technique will result in radiographic incorporation of the bone grafting and reduction of pain.

## Materials and methods

This retrospective study was approved by the institutional ethics committee and all participants and/or their caregivers gave informed consent for participation. All children and adolescents (< 18 years of age) who underwent treatment of idiopathic advanced elbow OCD (grade 3 or 4 according to Nelson [26]) using our technique described below between 2016 and 2018 were invited for a single clinical and radiologic follow-up examination at a minimum of one year after the intervention. The technique is reserved for advanced cases which show detached, unstable fragments in radiographs and MRI/CT (grade 3), or loose bodies (grade 4). All cases older than 18 years of age, those with posttraumatic osteochondral lesions and those with incomplete documentation were excluded. All patients were operated by a single fellowship-trained pediatric upper limb surgeon.

The included patients underwent clinical examination (elbow and forearm motion, stability, tenderness radial head/capitellum); furthermore, anterioposterior (AP), lateral, and AP radiographs in 45° elbow flexion were obtained in a standardized manner. Magnetic resonance imaging (MRI) sequences were obtained preoperatively and during the postoperative course. The following postoperative outcome scores were obtained: pain on the numeric rating scale (NRS; 0–10), Pediatric Outcome Data Collection Instrument (PODCI; parent-reported version; 0–100 [27]), Mayo Elbow Performance Score (MEPS, 0–100 [28]), and Timmerman-Andrews Score (TIMM, 0–200 [29]). The PODCI is a scale assessing upper extremity function, transfers and mobility, physical function and sports, comfort, happiness and satisfaction, and expectations for treatment in children. Both parent and adolescent self-report were developed and showed a high reliability and validity [27]. The Timmerman-Andrews Score, which was originally developed for cases with posttraumatic arthrofibrosis, is frequently beeing used in immature cohorts with elbow OCD [16].

The radiologic evaluation aimed to include the following pre- and postoperative OCD features: grade according to Minami [30], Berndt and Harty [31], and Nelson [26]; growth plate status (open/closed); AP and lateral diameter (in mm); AP width and depth (in % of total epicondylar diameter); congruency of the subchondral bone (flattened, concave, convex); sclerosis (none/yes); dissection (none, partial, complete/loose body); radiolucency (reduced, increased, normal), and fragmentation (none, heterogeneous area, distinct fragmentation). The MRI-related OCD healing after surgery was graded according to Roberts et al. (total score, 0–4) [32].

### Surgical technique

**Fig. 1:**
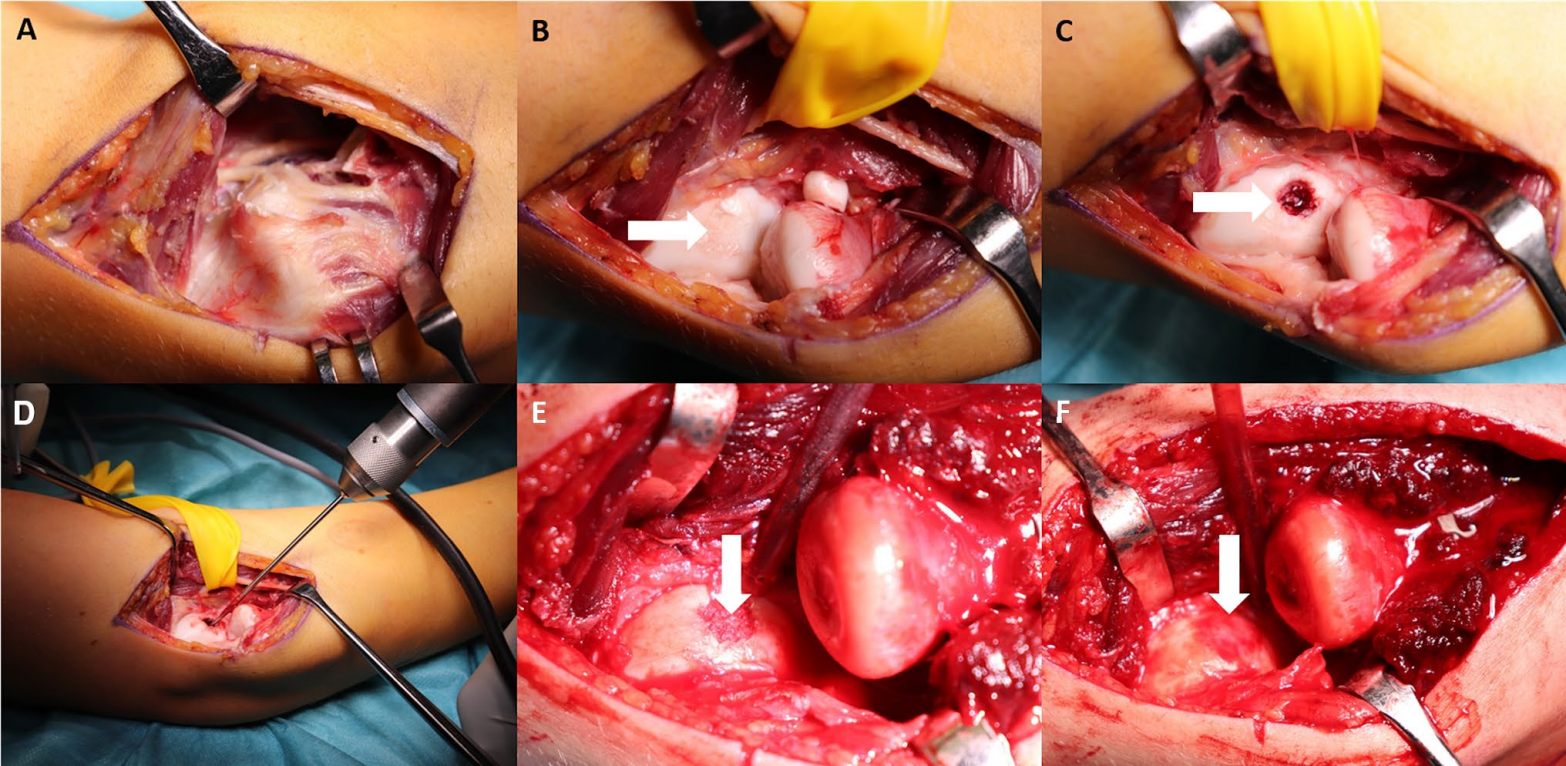
The surgical approach (**a**) and technique are presented. After joint inspection and removal of a loose body (**b**), the defect area is thoroughly curretted to remove all unstable tissue (**c**, *arrow*). Then, antegrade drilling is performed and the cancellous bone graft is tightly impacted (**d**, **e**, *arrow*). Finally, the scaffold is cut to fit the defect, injected with bone marrow aspirate, and applied over the defect (**f**, *arrow*). It is held stable using fibrin glue

**Fig. 2:**
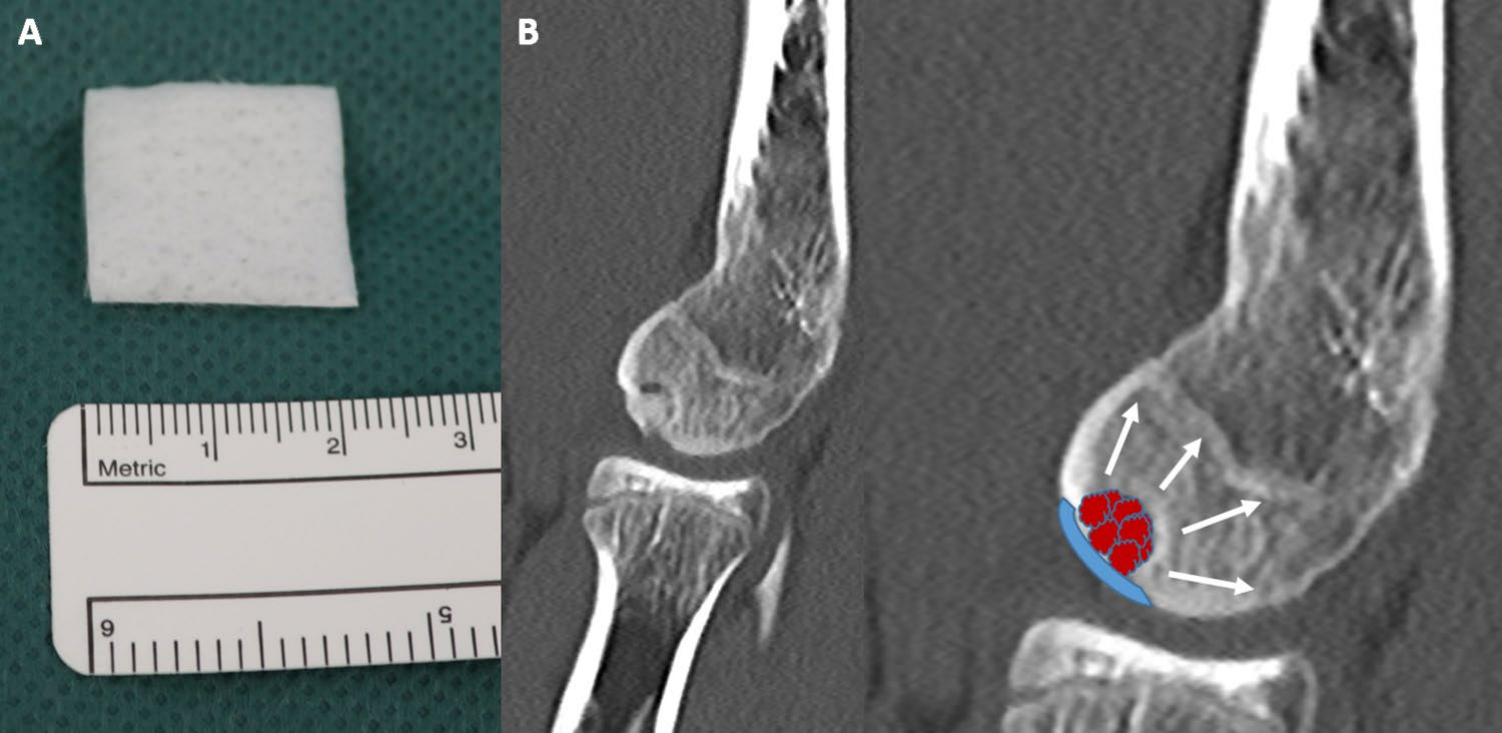
The HYALOFAST® membrane (2 × 2 cm) is shown (**a**). A schematic drawing illustrates the important steps, such as antegrade drilling of the capitulum, to enhance regeneration (white arrows), impaction of cancellous bone graft and application of the membrane (**b**)

The surgical approach is similar to the one described previously [33]. Briefly, a lateral Kocher approach to the elbow patient is performed with the patient lying in the supine position (Fig. 1a). The fascia is divided and the interval between the extensor digitorum communis and extensor carpi ulnaris is separated to expose the joint capsule. The capsule is then divided and the lateral collateral ligaments are released whenever proper inspection of the capitellum is impaired. The capitellum is thoroughly assessed to search for the affected OCD area, in particular, i.e. cartilage softening, fissures or detached fragments (Fig. 1b, arrow). Any loose boodies are removed instantly, and the radial head is inspected for any concomitant alterations. Then, the OCD defect is debrided (Fig. 1c, arrow). For this reason, the cartilage rim is trimmed to stable edges using the scapel. The subchondral bone layer is sharply curretted to remove the superficial fibrinous layer and all bone material of inferior quality. After the curretage, the OCD area is circumferentially drilled in an antegrade fashion at low drill speed until bleeding is visible (Fig. 1d). Then, autologous cancellous bone as well as bone marrow is harvested from the iliac crest. The cancellous bone is impacted in the defect (Fig. 1e, arrow). It is mandatory to avoid overstuffing of the defect since the reconstructed joint surface should eventually have the same level like the surrounding healthy cartilage (Fig. 1f). Finally, the scaffold (HYALOFAST®; Anika Therapeutics S.r.L., Italien; Fig. 2) is cut to fit the defect size, injected with bone marrow and layed over the defect (Fig. 1f, arrow). The membrane is structured as a 3-dimensional matrix which releases hyaluronic acid during its degradation. Fibrin glue is used to stabilize the membrane (Fig. 1f). The lateral collateral ligaments are repaired with non-absorbable sutures whenever necessary. After capsular repair and skin closure, a long arm cast is applied for 3 weeks. Thereafter, protected exercises are allowed using an elbow orthosis (Epico ROMs; medi GmbH & Co. KG; Bayreuth, Germany) and occupational therapy. Full loadbearing and manual activity is allowed 3 months postoperatively. Sports participation is resumed after 6 months.

### Statistical analysis

A descriptive calculation of means, minimum and maximum of the main outcome parameters and demographic details were performed. All analyses were performed using SPSS v23.0.

## Results

This series included 5 adolescent patients (3 males, 2 females) with a mean age of 13.4 years (range, 12–15) at the time of surgery and 17.3 years at final follow-up (range, 15–20). Hence, the mean follow-up period was 34 months (range, 24–45). Further demographic details are listed in Table 1. Two cases received diagnostic arthroscopy prior to the intervention in the same session to verify fragment instability.

**Table 1:** Demographic details

Case	Sex	Side	Age	BMI	Minami	Berndt and Harty	Nelson	Sports Participation	Additional Surgery
1	F	R	13	18	III	IV	IV	Yes	None
2	F	L	14	19	II	III	III	Yes	None
3	M	R	12	-	I	II	III	Yes	Arthroscopy
4	M	R	15	20	III	III	III	Yes	Arthroscopy
5	M	R	13	19	III	IV	IV	Yes	None

M, male; F, female; R, right; L, left; OCD, osteochondrosis dissecans; BMI, body mass index

All patients reported an NRS score of 0. Elbow flexion and extension improved from a mean of 132° (range 110–140) and − 6° (range – 15 to 0) preoperatively to 135° (range 125–140) and 0° (range 0–0) at latest follow-up. The forearm motion arc improved from a mean of 164° (range 120–180) to 176° (range 160–180) after surgery. The mean total TIMM was 189 (range 165–200). TIMM subscales indicated good to excellent outcomes for both subjective (mean, 91; range 65–100) and objective (mean 98; range 90–100) measures. The mean postoperative MEPS was 94 (range 70–100). The mean postoperative PODCI Global Functioning Scale was 92 (range 83–98; normative score 47; range 35–55). All detailed PODCI scales and scores are shown in Table 2.

**Table 2:** Clinical outcome measures

Case	NRS	Timmermann			MEPS	PODCI					
		Total	Subjective	Objective		Upper Extremity Scale Standardized Mean	Transfer and Basic Mobility Scale Standardized Mean	Sports and Physical Functioning Scale Standardized Mean	Pain or Comfort Scale Standardized Mean	Happiness Scale Standardized Mean	GLOBAL FUNCTIONING SCALE Mean of Standardized Means
1	0	180	90	90	100	96	100	83	78	100	89
2	0	165	65	100	70	92	97	85	61	60	83
3	0	200	100	100	100	100	100	100	93	80	98
4	0	200	100	100	100	100	100	97	85	100	96
5	0	200	100	100	100	96	94	92	100	85	95
**Mean**	**0**	**189**	**91**	**98**	**94**	**97**	**98**	**91**	**83**	**85**	**92**

*NRS* Numeric rating scale, *MEPS* Mayo Performance Elbow Score, *PODCI* Pediatric data outcome instruments

**Fig. 3:**
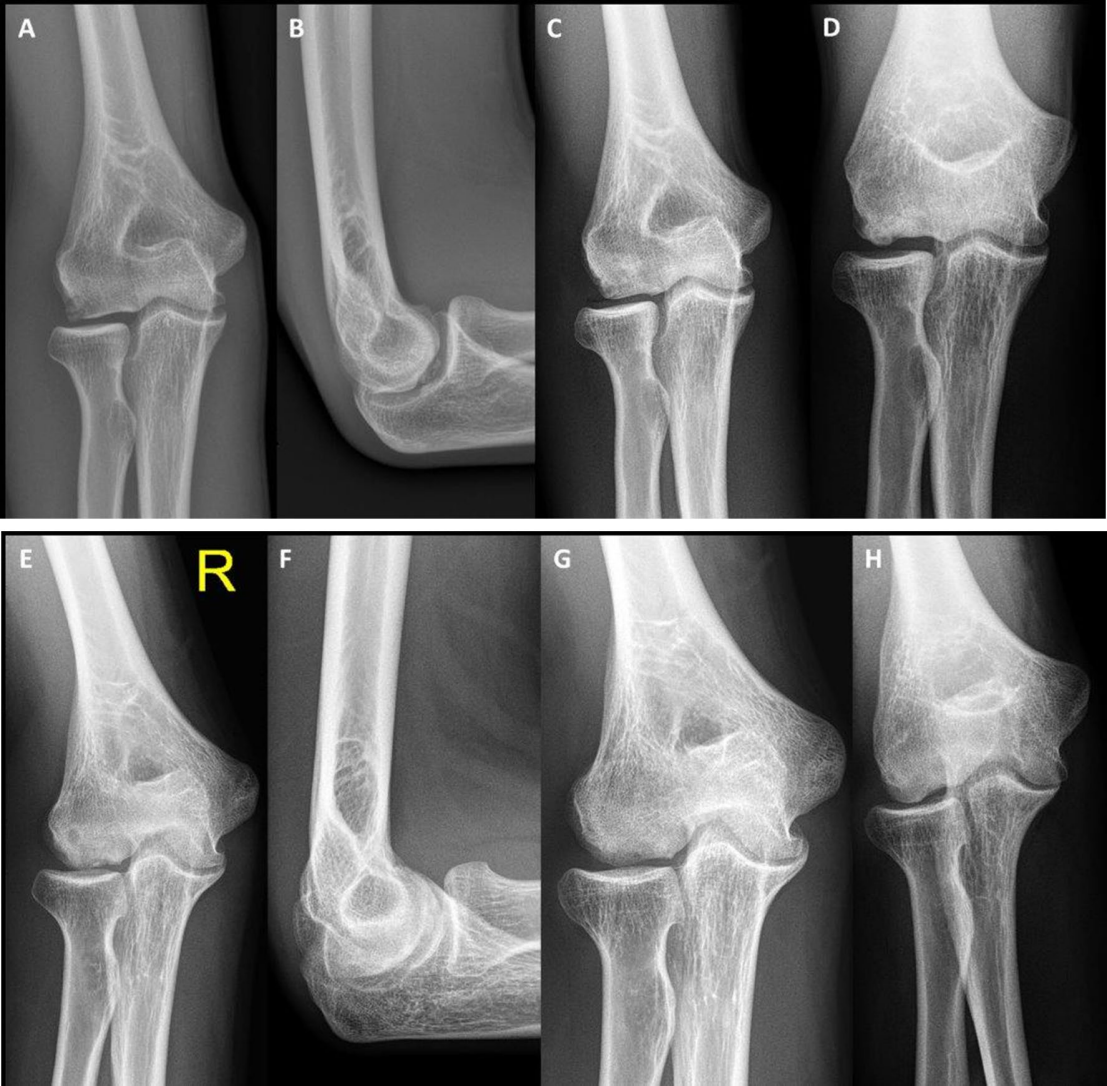
Two OCD cases are presented. Case 4 revealed a lateral sided defect (**a**) with a detached fragment (**b**) which showed good resolution after surgery (**c**). However, 45° AP views confirmed some irregularities, likely resembling the cancellous graft (**d**). Case 5 showed a severe affection of the entire condyle (**e**, **f**) but also healed well without any residual symptoms. Postoperative images revealed a homogeneous situation, however, the subchondral area was still concave and slightly hypointense (**g**, **h**)

The radiographic parameters showed a mean decrease of the affected OCD area of 35% in width (range − 27 to 100%) and 52% in depth (range, 4–100%), respectively. Cases no. 4 and 5 (Fig. 3) showed complete resolution of the reconstructed OCD area while the other cases showed residual findings, such as reduced bone transparency (*n* = 3). A detailed summary of the radiologic findings is highlighted in Table 3.

**Table 3:** Radiographic Outcome and Details

Case	Initial Physis Status	Final Physis Status	Width Improvement	Depth Improvement	Final Congruency	Final Sclerosis	Final Dissection	Final Transparency	Final Fragmentation
1	Closed	Closed	− 9%	**4%**	Convex	**No**	**No**	**Normal**	**No**
2	Closed	Closed	**10%**	**7%**	**Convex**	**No**	No	Reduced	No
3	Open	Almost closed	– 27%	**47%**	Convex	**No**	No	Reduced	No
4	Closed	Closed	**100%**	**100%**	**Convex**	No	**No**	**Normal**	No
5	Open	Closed	**100%**	**100%**	Concave	No	No	**Normal**	No

Improvements are highlighted in bold

The MRI findings showed a mean Roberts score of 2.0 (range 1–3) on the scale of 0–4 (Table 4).

**Table 4:** MRI findings

Case	Surface Integrity and contour	Cartilage signal in graft region	Cartilage thickness	Changes in underlying bone	Total score*
1	0	0	0	1	1
2	0	0	1	1	2
3	0	1	1	1	3
4	0	1	1	0	2
5	0	0	1	1	2

1 = normal or near normal, 0 = abnormal

*Aggregate score (4 points maximum possible)

## Discussion

Elbow OCD in children and adolescents is a frequent pathology with a high risk for morbidity. In cases with moderate to severe disruption of the subchondral bone, restoration of this affected area and the altered cartilage layer is mandatory. The aim of this case-series was therefore to present our preliminary findings of a new technique which addresses both the degenerated cartilage and bone area.

Early cases of OCD have been shown to sufficiently respond to activity modification and rest [9]. Niu et al. reported that > 50% of stable OCD cases resolved after a mean period of 8.3 months [34]. Predictors for healing were a smaller OCD area and lack of cyst-like lesions [34]. In turn, a large study of 245 elbows has shown that, among 48 non-surgical cases, radial head enlargement and advanced skeletal age were predictors for a lack of spontaneous healing [35].

In cases where OCD still progresses to increased capitellar involvement, drilling/microfracture or fragment refixation has shown differring results depending on the state of the disease. Matsuura et al. have shown that arthroscopy with concomitant drilling provided enduring and independent results in the long-term indepent of the lesion size [13]. However, in their study, only 6/23 patients (baseball players) had large OCD lesions (defect size > 60%) [13]. These results contrast the findings of Ueda et al. who provided evidence that larger OCD lesions, which were simply resected, progressed to osteoarthritis after a mean of 8 years postoperatively [10]. Moreover, another study reported that just 71% of cases had either clinical or radiographic resolution (50% complete resolution on MRI, 62% clinically non-tender) after drilling of grade IV lesions [12]. Despite a relatively high return-to-any-sports-rate, 43% still reported mild elbow pain and persisting mechanical symptoms after this surgery [12]. In contrast, Takeba et al. and Hennrikus et al. reported that, in unstable but still in situ lesions, fragment refixation can provide a reasonable healing percentage with good outcomes for the majority of children [14, 15].

As a consequence of the findings described above, many authors tend to replace the subchondral, malvascularized bone in advanced OCD stages (e.g. grades III to IV) using osteochondral bone grafts [16, 17, 36, 37]. This can provide additional healthy hyaline cartilage as new joint surface tissue, either transplated as a single bone plug or mosaicplasty. A host of different reports exist showing that favorable results can be achieved with the OAG technique. Bae et al. reported significant improvements in elbow motion, 93% graft incorporation into the recipient site, and no complications thereafter [16]. As compared to Bae et al. and most other authors who used femoral trochlear autografts, Sato and colleagues reported excellent outcomes after transplantation of costal OAG [17]. Oshiba et al., who used multiple bone plugs for mainly early lesions (ICRS grade I and II; Minami stage I and II) reported complete radiological healing in 8 of 11 cases; however, healing needed up to 24 months to become evident in MRI [16]. Another study has, however, shown that large-size defects, which span from the lateral to the central articular portion, may not heal as well as central lesions after OAG transplantation [36, 37].

The harvest of autologous bone plugs is, however, not without the risk of complications and morbidity [21]. Up to 13% of patients reported knee symptoms after OAG harvest. Therefore, many patients, especially those involved in sports participation, are in our experience rather reluctant to agree to harvest tissue of their healthy knee. This was one of the reasons for our group to look for alternative techniques to repair the capitellum.

We thus aimed to repair the defect area by thorough debridement (curretage of unstable, softened bone tissue), drilling to enhance re-vascularization, and autologous iliac crest cancellous bone graft infill. The graft harvest is usually very well tolerated since it is performed via a 1 cm mini-incision with a bone punch. The donor site morbidity in this area is more appealing for many patients as compared to the knee joint. The application of the scaffold membrane is easy to do, and already proved to be efficacious in other past reports [23–25]. We additionally prefer to add iliac crest blood to enhance stem-cell-related ingrowth.

**Fig. 4:**
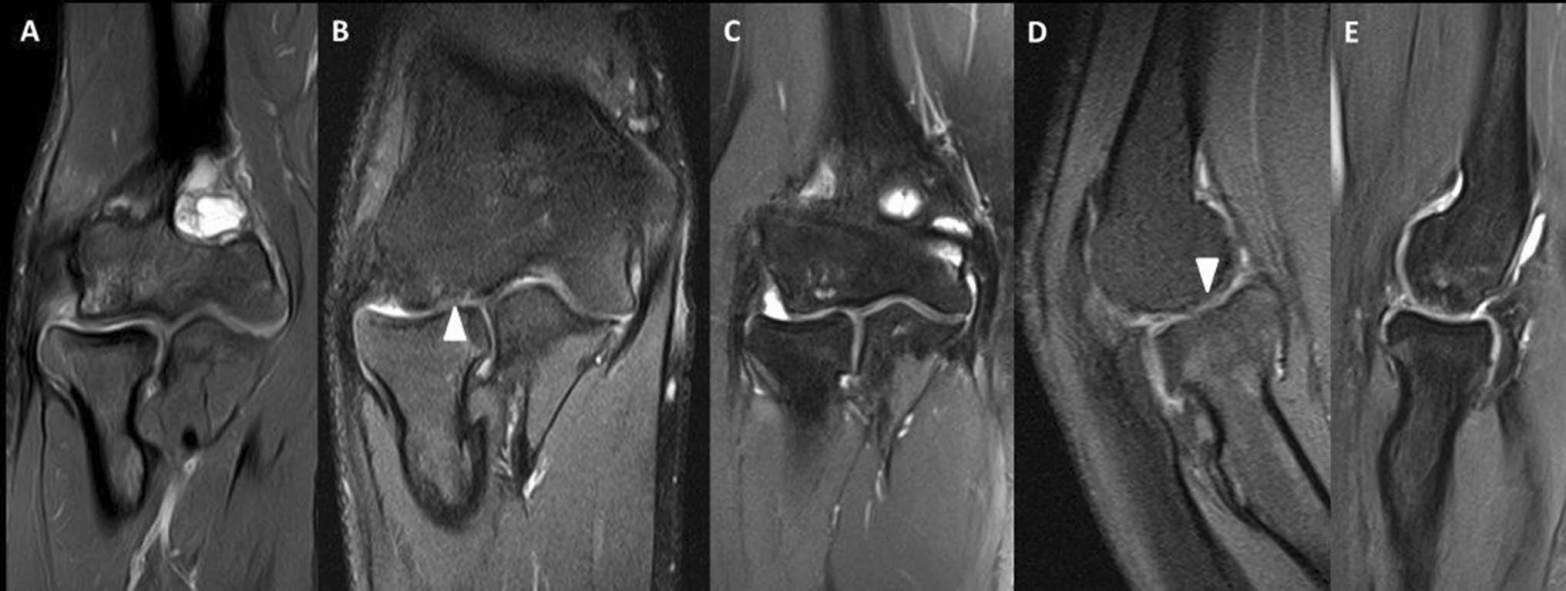
The postoperative MRI sequences of cases 4 (**a**), 5 (**b**, **c**) and 2 (**d**, **e**) are shown. Reduction of OCD-related edema is visible in all cases. Fibrous regenerate cartilage is highlighted in B and D (*arrowhead*)

The results of our technique were so far quite favorable with regard to clinical improvement. All five patients reported no symptoms at all. One case, a professional skier, experienced residual pain during heavier activities which eventually resolved two years after the surgery. The good outcomes are supported by good to excellent Mayo Elbow Performance, Timmerman Andrews and PODCI scores, respectively. In contrast, radiological findings were moderate. We observed an expected decrease of the OCD area at follow-up, but no full radiologic healing was evident in native radiographs in 3/5 cases. This was, however, not surprising, since the cancellous bone graft may not have the same structure and architecture of the native capitellar bone. However, several partial improvements, such as a changes of the subchondral congruency from “flattened” to “convex” (in 2 cases), reduction of sclerosis (in 2 cases) and normalization of transparency and fragementation (in 1 case each) were observed. In regard to MRI findings, previous studies have shown that signs of the disease may still be visible up to 24 months after surgery [36]. Our postoperative MRIs showed lack of subchondral and capitellar bone marrow edema with, however, expected irregularities next to the healthy hyaline cartilage as a sign of fibrous regenerate tissue in the scaffold area (Fig. 4).

The only comparable study, published by Guerra et al., reported on the outcomes following bone marrow-derived cell transplantation in 3 adolescents [22]. In this technique, platelet-rich fibrin was used to enhance local regeneration after clearing of the lesion. All patients reported clinical improvements and resumed sports after 9 months. MEPS improved from 78.3 to 93.3 and the Oxford Elbow Score from 40.0 to 47.6. As seen in our current study, continuous healing with regeneration tissue was seen over time.

The limitation of this study is the small study cohort and the preliminary, mid-term character of our findings. Furthermore, no alternative technique was included to serve as controls and the radiographs were rated by a single observer (treating surgeon). It remains to be determined whether the results are durable over a longer period and whether radiographic healing may advance over time. We did not include a standardized MRI protocol in our follow-up evaluation and hence, MRIs were obtained at different time points (10–32 months) postoperatively.

In summary, the findings of our novel technique showed promising results in adolescent patients with severe OCD. Due to its relevant advantages, such as the possibility to reconstruct the defect height exactly as needed longer follow-up will be pursued to confirm its suitability as an alternative technique to OAG.
